# Pembrolizumab‐induced myasthenia gravis with myositis and presumable myocarditis in a patient with bladder cancer

**DOI:** 10.1002/iju5.12128

**Published:** 2019-10-30

**Authors:** Maki Todo, Gou Kaneko, Suguru Shirotake, Yuki Shimada, Shintaro Nakano, Takashi Okabe, Shiho Ishikawa, Masafumi Oyama, Koshiro Nishimoto

**Affiliations:** ^1^ Department of Pharmacy Saitama Medical University International Medical Center Hidaka Saitama Japan; ^2^ Department of Uro‐Oncology Saitama Medical University International Medical Center Hidaka Saitama Japan; ^3^ Department of Palliative Care Internal Medicine Saitama Medical University International Medical Center Hidaka Saitama Japan; ^4^ Department of Cardiology Saitama Medical University International Medical Center Hidaka Saitama Japan

**Keywords:** myasthenia gravis, myocarditis, myositis, pembrolizumab

## Abstract

**Introduction:**

Pembrolizumab cause immune‐related adverse events. We herein report a case of advanced bladder cancer, who treated with pembrolizumab and exhibited intriguing clinical course.

**Case presentation:**

A 63‐year‐old man with bladder carcinoma was treated by radical cystectomy, however, the bladder carcinoma recurred and invaded to the rectum. He was treated by combination therapy using gemcitabine and cisplatin, which were not effective for the tumor. He subsequently underwent treatment with pembrolizumab. In several 30 days, he suffered from the symptoms of myasthenia gravis. Serum levels of creatine kinase, its isozyme creatine kinase‐myocardial band, and troponin I were elevated. Electrocardiography showed a right bundle branch block. These findings suggested that he was myasthenia gravis with general myositis and presumable myocarditis. Oral prednisolone administration significantly attenuated these findings. The tumor drastically shrunk only by the single injection of pembrolizumab.

**Conclusion:**

Early intervention with corticosteroid was effective for neuromuscular complications due to pembrolizumab.

**Abbreviations & Acronyms**
AChR Ab: acetylcholine receptor antibody
CK: creatine kinase
CT: computed tomography
ECG: electrocardiography
irAEs: immune‐related adverse events
MB: myocardial band
MG: myasthenia gravis
MRI: magnetic resonance imaging
MuSK Ab: muscle‐specific kinase antibody
PD‐1: programmed cell death 1
SIMC‐Uro: Uro‐oncology Saitama Medical University International Medical Center
UC: urothelial carcinoma

**Keynote message**

Immunosuppressive treatment, for example, with corticosteroids, must be considered when we notice the sign of MG with myositis arises in a patient under treatment with a PD‐1 inhibitor. Immune‐mediated neuromuscular side effects vary in presentation, and differ from their idiopathic counterparts.

## Introduction

Pembrolizumab, a humanized monoclonal antibody that inhibits PD‐1 receptors, is utilized as a second‐line therapy for advanced UC, which gives complete remission in approximately 7% of patients.^1^ However, it is reported that pembrolizumab causes several irAEs including myositis and MG.^2^ Here, we report a case of pembrolizumab‐induced MG with myositis in a patient with bladder cancer, who accomplished complete remission.

## Case presentation

**Figure 1:**
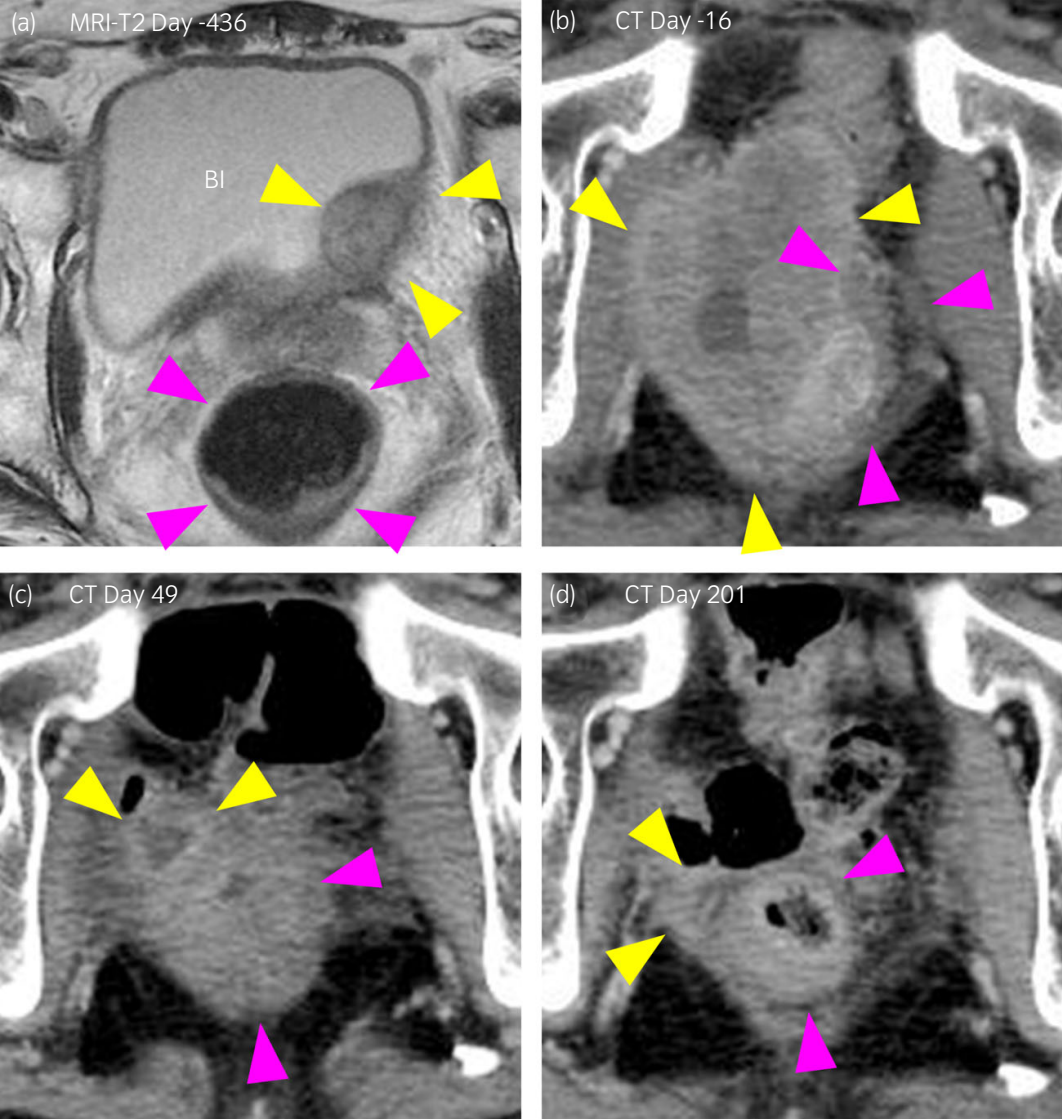
Imaging study of the case. (a) A T2‐weighted image of MRI on day 436. (b) CT performed before pembrolizumab treatment on day 16. (c, d) CT after pembrolizumab treatment on day 49 and 201, respectively. Yellow and pink arrowheads in panels (a–d) indicate bladder tumor (clinical T2–T3) and the rectum, respectively.

A 63‐year‐old Japanese man (SIMC‐Uro #9950, a unique non‐sequential patient control number in the department of SIMC‐Uro) visited a nearby clinic due to intermittent macroscopic hematuria. He had a medical history of mild diabetes mellitus and surgery for colon carcinoma. Physical and laboratory findings were within normal limits except for the presence of microhematuria at the clinic. Cystoscopy revealed a broad‐base non‐papillary bladder tumor. CT (#1, Table S1) and MRI (#1, Fig. 1a) revealed a bladder tumor invading perivesical tissue without apparent metastases (cT3N0M0). He underwent transurethral resection of the tumor, and the pathological diagnosis of the resected specimen was UC that invaded sub‐epithelial connective tissues (pT1).

He was referred to the SIMC‐Uro for the treatment of the invasive bladder tumor. Physical and laboratory findings were within normal limits (height 175 cm, body weight 63 kg). CT #2 at the SIMC detected no metastatic lesions. He underwent three cycles of monthly neoadjuvant chemotherapy using gemcitabine (1000 mg/m^2^, day 1, 8, and 15) and cisplatin (70 mg/m^2^, day 2), followed by radical cystectomy and extended pelvic lymph node dissection. Histopathological examination detected high‐grade UC in the previously resected portion even after chemotherapy, however, no cancer cells were detected in the surgical margin of the bladder specimen (pT1N0).

CT #5 which was performed 266 days after radical cystectomy, identified cancer recurrence in the pelvic wall (50 mm in length) which invaded to the rectum (Fig. 1b). Transrectal biopsy of the tumor pathologically conformed the recurrence of the bladder cancer. Irrespective of two cycles of salvage chemotherapy using the same regimen as the neoadjuvant setting, the recurrent tumor significantly enlarged to 81.6 mm (CT #5, Fig. 1b).

**Figure 2:**
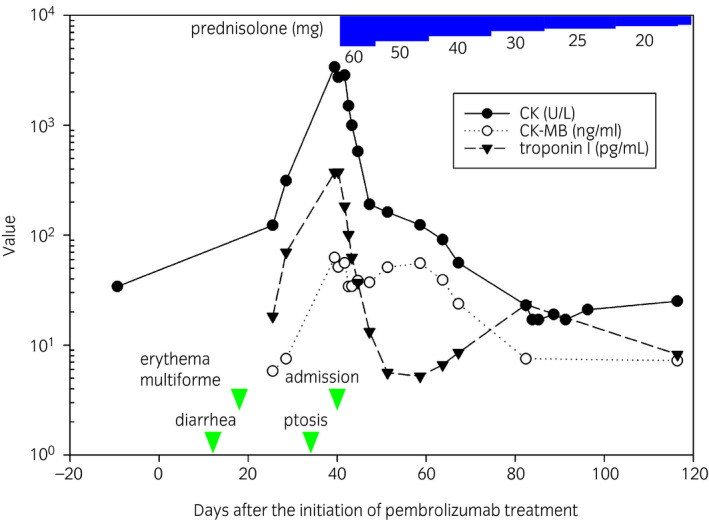
Clinical course of the case. Clinical course of the case after pembrolizumab treatment. Green arrowheads indicate occurrence date of irAEs and time of hospitalization. Laboratory data of CK, CK‐MB, and troponin I and the dose of prednisolone.

**Figure 3:**
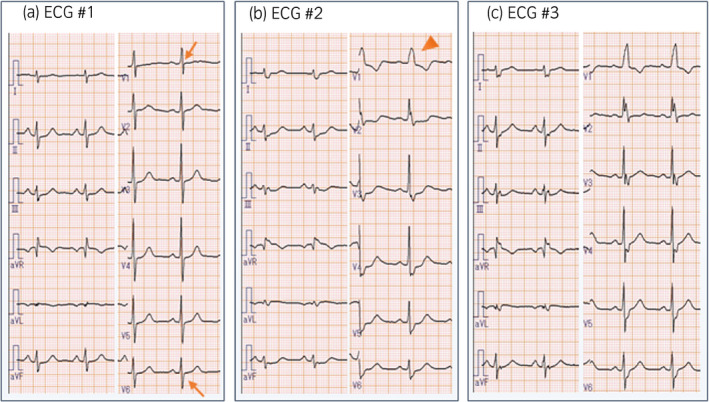
Changes in electrocardiographic findings. The electrocardiogram at baseline suggested undetermined axis and mild right ventricular loading (panel a, arrows). The complete right bundle branch block (QRS duration: 148 milliseconds developed, panel b, arrowheads). Although the complete right bundle branch block was performed in the remote period after steroid therapy, the QRS duration was slightly shortened to 134 milliseconds (panel c).

The patient initiated treatment with tri‐weekly 200 mg of pembrolizumab injection 283 days after radical cystectomy. Of note, 9 days before the treatment, the case complaint of any symptom except for mild diarrhea (Grade 1 in Common Terminology Criteria for Adverse Events version 4.0 [https://www.eortc.be/services/doc/ctc/]) due to unknown reason. At the day of Grade 1‐diarrhea, laboratory data including serum CK level (CK: 34 [normal range: <244] U/L) (Fig. 2) and ECG (#1, Fig. 3a) were normal. From 12 to 34 days after the administration of pembrolizumab, he serially complained of Grade 3‐diarrhea, Grade 3‐erythema multiforme with pruritus and left ptosis with diplopia. Our comprehensive management team consisting of pharmacists, nurses, cardiologists, and neurologists intervened to this case. Laboratory examination showed significantly elevated CK (3385 U/L), its isozyme CK‐MB (62.5 [<5] n/mL), and troponin I (372.1 [<26.20] pg/mL) (Fig. 2). ECG#2 findings revealed a right bundle branch block (Fig. 3b) which was not observed at ECG#1 (Fig. 3a) although echocardiographic findings were normal. Overall, the ECG, echocardiography, and laboratory data suggested that he had mild myocardial damage with conduction disturbance. MG and myositis due to autoimmune abnormality was unlikely, because their specific examinations including repetitive stimulation test, tensilon test, electromyography as well as serum measurements of anti‐AChR Ab and anti‐MuSK Ab were all negative. Consequently, the case was diagnosed as MG with myositis with systemic myopathy involving the cardiac and facial muscles as an irAE.

He initiated 60 mg/day of prednisolone (1 mg/kg, Fig. 2). Serum levels of CK, CK‐MB, and troponin I as well as symptoms including diarrhea and ptosis gradually improved. Although the conduction disturbance remained in the remote period, the patient demonstrated no impairment of ventricular function throughout the whole observation period. Interestingly, the tumor significantly shrunk to 25 mm only 49 days after the single pembrolizumab administration (CT #6, yellow arrowheads in Fig. 1c). The dose of prednisolone was gradually reduced to as shown in Figure 2. The tumor remained small without any treatment after a single injection of pembrolizumab as of 201 days after the pembrolizumab injection (CT #8, Fig. 1d). Prednisolone was withdrawn 362 days after the pembrolizumab injection (321 days treatment with prednisolone). Consequently, irrespective of severe neuromuscular complications, this case accomplished putative pathological complete remission by the single injection of pembrolizumab.

## Discussion

We encountered a patient with advanced UC, who developed neuromuscular complications including MG, myositis due to irAE by pembrolizumab treatment. These side effects resolved by early introduction of prednisolone therapy, otherwise the case possibly deceased especially by heart failure due to myocarditis.

It is reported that myositis and MG as an irAE are rare (<1%),^2^, ^3^ most of which occur within 2 months after the initiation of pembrolizumab treatment.^2^ Patients with these complications typically show a high level of serum CK due to the destruction of myocytes. Autoimmune myositis and MG are generally diagnosed based on criteria including typical symptoms (e.g. diplopia and ptosis) and laboratory tests (e.g. high levels of serum anti‐AChR Ab and anti‐MuSK Ab).^4^, ^5^ However, as shown in the current case, clinical characteristics and pathophysiology of neuromuscular irAE seems to be different from those due to autoantibodies.^2^ Notably, cardiac complications occurred in more than 30% of patients diagnosed with neuromuscular irAE,^2^ hence, the screening of cardiovascular disease is mandatory for these patients early intervention using corticosteroids can avoid lethal myocardial damage in cases with abnormal ECG.^6^

From the course of the current case, we concluded that (i) regular screening using CK, CK‐MB, and troponin I is needed for early detection of neuromuscular complication, (ii) cardiac complications must be identified by repetitive ECG and echocardiography to avoid lethal cardiac damage by early intervention, and (iii) comprehensive management team play a pivotal role for these early detection and intervention. Further accumulation of case reports is needed to sophisticate cancer treatment using immune checkpoint inhibitors.

## Conflict of interest

The authors declare no conflict of interest.

## Supporting information


**Table S1.** Clinical course of the case.
